# Supplementation of *Bacillus coagulans* and Tributyrin to Danzhou Chickens: Effects on Growth Performance, Antioxidant Status, Immune Response, Intestinal Health, and Cecal Microbiome

**DOI:** 10.3390/ani15233428

**Published:** 2025-11-27

**Authors:** Xilong Yu, Fei Xu, Dexin Zhao, Haoliang Chai, Yang Yu, Weiqi Peng, Liangmei Xu, Hongzhi Wu

**Affiliations:** 1 Tropical Crops Genetic Resources Research Institute, Chinese Academy of Tropical Agricultural Sciences, Haikou 571101, China; 2 College of Animal Science and Technology, Northeast Agricultural University, Harbin 150030, China; 3 Junan Agriculture and Rural Bureau, Linyi 276600, China

**Keywords:** Danzhou chicken, *Bacillus coagulans*, tributyrin, growth performance, immune function, gut microbiota

## Abstract

To investigate the effects of *Bacillus coagulans* (BC) and tributyrin (TB) on Danzhou chickens, a 2 × 2 factorial design was adopted. A total of 480 chickens were randomly assigned to four dietary treatments, consisting of two BC levels (0 and 1.5 g/kg) and two TB levels (0 and 1.0 g/kg), for a 35-day trial. The results showed that supplementation with BC or TB alone significantly increased the average daily gain (ADG), serum immune parameters (immunoglobulin A, immunoglobulin G, and anti-inflammatory cytokines interleukin-4 and interleukin-10), total antioxidant capacity, and catalase activity, while reducing pro-inflammatory cytokine levels and the feed-to-gain ratio (*p* < 0.05). In addition, individual supplementation with BC or TB also enhanced digestive enzyme activities in the intestine, increased villus height in the small intestine, and optimized the structure of the cecal microbiota (*p* < 0.05). More importantly, significant synergistic interactions between BC and TB were observed across multiple parameters (*p* < 0.05). Combined supplementation further increased ADG, serum immunoglobulin M levels, superoxide dismutase activity, lipase activity in the ileum, and villus height in the jejunum (*p* < 0.05). Meanwhile, the combined supplementation also significantly elevated the abundance of beneficial bacteria such as *Bacteroides*, *Eubacterium_brachy_group*, and *Negativibacillus*. In conclusion, supplementation with BC or TB effectively improved the growth performance, antioxidant status, immune function, intestinal morphology, and cecal microbiota composition of Danzhou chickens, and the combined supplementation demonstrated superior effects compared to individual supplementation.

## Simple Summary

The extensive use of antibiotics has raised concerns about drug residues in meat and the emergence of antimicrobial resistance, posing substantial risks to public health. This situation highlights the urgent need to develop safe and effective antibiotic alternatives to support sustainable poultry production. *Bacillus coagulans* (BC) is a safe feed additive known to enhance growth performance, antioxidant capacity, and intestinal microbial composition in poultry. Tributyrin (TB) provides a controlled release of butyrate in the small intestine, thereby improving growth performance, digestive enzyme activity, and intestinal health in broilers. Therefore, the combined supplementation of BC and TB may represent a promising strategy for promoting growth. The results of this study demonstrate that their combined application produces a marked synergistic effect, leading to substantial improvements in multiple key physiological and productive traits in Danzhou chickens. These findings offer a strong theoretical basis for the practical use of these additives in poultry production.

## 1. Introduction

Poultry constitutes a major source of high-quality protein in the human diet and plays a vital role in ensuring global food security and nutritional adequacy [1]. Driven by rapid population growth and rising income levels, global demand for poultry meat and eggs continues to increase, thereby accelerating the shift toward intensive broiler production to meet expanding food requirements. In intensive farming systems, commercial broilers exhibit short rearing cycles and rapid growth rates, which may impose additional physiological stress on the intestinal tract and predispose birds to gastrointestinal disorders [2]. Historically, broiler production has relied heavily on antibiotics to enhance growth performance by inhibiting bacterial proliferation or directly eliminating pathogenic microorganisms [3]. However, extensive use of antibiotics has been shown to result in problems such as drug residues in meat and the emergence of antimicrobial resistance among pathogenic bacteria, thereby posing potential threats to human health [4]. Consequently, there is an urgent need to develop safe and effective alternatives to antibiotics to ensure the sustainable development of the poultry industry.

Probiotics, administered as live microbial feed supplements, can enhance growth performance and overall health in poultry and have emerged as promising alternatives to antibiotic-based interventions [5]. *Bacillus coagulans* (BC) is a Gram-positive probiotic capable of producing lactic acid and forming non-pathogenic spores. Owing to their high tolerance to acidity, elevated temperatures, and pressure, these spores enable BC to survive gastric acid and colonize the small intestine, thereby promoting the intestinal microbial balance [6,7]. Previous studies have shown that BC is a safe and suitable strain for feed supplementation, effectively enhancing poultry growth performance, antioxidant capacity, and intestinal microbiota composition [8]. Furthermore, this bacterium can improve feed utilization efficiency by secreting various digestive enzymes [9]. Butyric acid, a short-chain fatty acid, has attracted considerable attention because of its beneficial effects on energy metabolism of intestinal mucosal cells and intestinal microbial balance [10]. Facilitated by butyrate transporters, butyric acid can be rapidly absorbed by intestinal epithelial cells [11]. However, due to its irritating pungent odor, it is not suitable for direct application in feed production. Tributyrin (TB), a butyrate derivative formed by the esterification of three molecules of butyric acid with one molecule of glycerol, possesses a pleasant fruity aroma [12]. When TB reaches the small intestine, lipases hydrolyze the glyceride to release butyrate, thereby preventing premature absorption in the upper digestive tract [13]. Previous studies have indicated that during the finishing phase of broilers, TB can replace enramycin and significantly improve growth performance, digestive enzyme activity, and intestinal health [12,14]. Moreover, data from trials involving yellow-feathered broilers have demonstrated that the combined dietary supplementation of 1 g/kg TB and BC exerts a more pronounced synergistic effect on intestinal morphology and microbial community [15]. Therefore, we hypothesize that the combined supplementation of TB and BC could represent a promising growth-promoting strategy.

Danzhou chicken is a native chicken breed from Hainan Province, China, characterized by strong adaptability, tolerance to coarse feeding, and excellent meat quality [16]. This study aims to investigate the effects of dietary supplementation with BC and TB, individually or in combination, on growth performance, antioxidant capacity, immune function, intestinal morphology, and gut microbiota in Danzhou chickens. The findings of this study provide meaningful theoretical insights into the mechanisms underlying intestinal health and growth performance in Danzhou chickens.

## 2. Materials and Methods

### 2.1. Ethics Statement

The management and care procedures for the animals used in this study were approved by the Institutional Animal Care and Use Committee of the Chinese Academy of Tropical Agricultural Sciences (Approval No.: CATAS-20241112-2).

### 2.2. Animals, Diets, and Experimental Design

The BC strain (viable count: 2 × 10^8^ CFU/kg) used in this trial was provided by Hubei Green Snow Biotechnology Co., Ltd. (Xianning, China). The TB (purity ≥ 65%) was supplied by Harbin Jinfulai Technology Development Co., Ltd. (Harbin, China).

This experiment followed a 2 × 2 factorial completely randomized design. A total of 480 healthy one-day-old female Danzhou chickens (24.63 ± 0.60 g) were sourced from a local Danzhou chicken breeding base (Hainan Rekeyuan Ecological Breeding Co., Ltd., Danzhou, China). All chickens were assigned to four dietary treatment groups based on body weight using a random number sequence generated by Excel software (version 2021), with six replicates (pens) per group and 20 chickens per replicate. The dietary treatments for each group were as follows: the control group (CK) received a basal diet; the experimental groups included the BC group (basal diet + 1.5 g/kg BC), the TB group (basal diet + 1.0 g/kg TB), and the BC × TB group (basal diet + 1.5 g/kg BC + 1.0 g/kg TB). Blinding was implemented during data collection and analysis, and the operators were unaware of the group assignments. The basal diet was purchased from Guangdong Hengxing Group Co., Ltd. (Zhanjiang, China), and its ingredient composition and nutritional levels are presented in Table 1. The metabolizable energy, lysine, and methionine levels of the diet were calculated based on the Chinese Feed Database (2023) [17]. Crude protein content was determined according to the Chinese National Standard GB/T 6432-2018 [18] using the Kjeldahl method and analyzed with an automatic nitrogen analyzer (Kjeltec 8400, FOSS, Horsens, Denmark). Crude fiber content was measured using a crude fiber analyzer (SLQ-6A, Shanghai XianJian Instruments, Shanghai, China) following the Chinese National Standard GB/T 6434-2022 [19]. Calcium and phosphorus contents were determined in accordance with GB/T 6436-2018 [20] and GB/T 6437-2018 [21], respectively, with phosphorus content analyzed using an ultraviolet-visible spectrophotometer (UV-6100, Shanghai Metash Instruments, Shanghai, China). The trial lasted for 35 days. During the experimental period, Danzhou chickens had free access to feed and water and were subjected to continuous 24-h artificial lighting with an intensity maintained at 20–30 lux. For environmental control, the temperature in the brooding house was initially set at 32 ± 1 °C for the first three days and then gradually reduced by 1–2 °C per week, stabilizing at 26 ± 1 °C by day 35. Meanwhile, relative humidity was controlled at 60 ± 5% using an automatic ventilation and humidity regulation system, and ammonia concentration was consistently kept below 15 ppm to maintain good indoor air quality.

### 2.3. Measurement of Growth Performance

On day 1 and day 35 of the formal trial, all chickens were weighed before morning feeding to record their initial and final body weights, respectively. Throughout the trial period, daily feed provision and residual feed quantities were recorded. Based on these data, the average daily gain (ADG), average daily feed intake (ADFI), and feed conversion ratio (FCR) were calculated.

### 2.4. Sample Collection

On day 35 of the trial, one Danzhou chickens close to the average body weight of each pen were selected and fasted for 12 h before sampling. Blood samples were first collected from the brachial vein under the wing into pre-labeled 10 mL centrifuge tubes, which were tilted and left to stand at room temperature. The samples were then centrifuged at 3000 rpm for 20 min at 4 °C. The resulting serum was transferred into 1.5 mL Eppendorf tubes and stored at −20 °C. Subsequently, the selected chickens were euthanized by intravenous injection of sodium pentobarbital (50 mg/kg body weight). After abdominal dissection, immune organs including the thymus, spleen, and bursa of Fabricius were excised and weighed to calculate the organ index (organ weight [g]/body weight [g]). Segments of approximately 2–3 cm in length from the duodenum, jejunum, and ileum were collected, gently rinsed with physiological saline, and fixed in 4% paraformaldehyde solution for subsequent intestinal morphological analysis. Meanwhile, the contents of the small intestine (duodenum, jejunum, and ileum) and the cecum were collected into sterile cryotubes, rapidly flash-frozen in liquid nitrogen, and subsequently stored at –80 °C for later analyses of digestive enzyme activities and gut microbiota composition.

### 2.5. Measurement of Serum Biochemical Indicators

Serum levels of total protein (TP), albumin (ALB), globulin (GLB), total cholesterol (TC), triglyceride (TG), high-density lipoprotein (HDL), low-density lipoprotein (LDL), urea, uric acid (UA), ammonia nitrogen (AN), non-esterified fatty acids (NEFA), and alkaline phosphatase (ALP) in Danzhou chickens were determined using specific assay kits (Zhong Sheng Bei Kong Bio-Tech Co., Ltd., Beijing, China) in combination with a biochemical autoanalyzer (Mindray BS-420, Wuhan Shengshida Medical Equipment Co., Ltd., Wuhan, China). All measurement procedures were strictly performed in accordance with the manufacturer’s instructions.

### 2.6. Serum Immune Indicators

Serum levels of immunoglobulin A (IgA), immunoglobulin G (IgG), immunoglobulin M (IgM), interleukin-1β (IL-1β), interleukin-6 (IL-6), tumor necrosis factor-α (TNF-α), interleukin-4 (IL-4), and interleukin-10 (IL-10) in Danzhou chickens were measured using commercial ELISA kits from Beijing Huaying Biotechnology Research Institute (Beijing, China) in combination with a microplate reader (DR-200Bs, Wuxi Huawei Delang Instrument Co., Ltd., Wuxi, China). The sensitivity, detection range, and intra- and inter-assay coefficients of variation for each indicator are detailed in Supplementary Table S1. All measurement procedures were strictly performed in accordance with the manufacturer’s instructions, and concentrations were determined based on calibration curves established using the standards provided with the kits.

### 2.7. Serum Antioxidant Index Measurement

Serum levels of superoxide dismutase (SOD), total antioxidant capacity (T-AOC), and catalase (CAT) in Danzhou chickens were measured using commercial assay kits (Beijing Huaying Biotechnology Research Institute, Beijing, China) with a microplate reader (DR-200Bs, Wuxi Huawei Delang Instrument Co., Ltd., Wuxi, China), following the manufacturer’s instructions strictly. The sensitivity, detection range, and intra- and inter-assay coefficients of variation for each indicator are detailed in Supplementary Table S2. All measurement procedures were strictly performed in accordance with the manufacturer’s instructions.

### 2.8. Digestive Enzyme Activity Assay

Approximately 0.3 g of intestinal content from the duodenum, jejunum, and ileum, respectively, was homogenized in a nine-fold volume of physiological saline (1:9, *w*/*v*) under ice-bath conditions using a TissueMaster^TM^ high-throughput tissue grinder (Beyotime, Shanghai, China). The homogenate was then centrifuged at 3000 rpm for 10 min at 4 °C. Subsequently, 50 μL of the resulting supernatant was collected for determining the activities of amylase, trypsin, and lipase. Assay kits were obtained from the Nanjing Jiancheng Bioengineering Institute (Nanjing, China), and all procedures were performed strictly according to the manufacturer’s instructions.

### 2.9. Intestinal Histomorphology

After 24 h of fixation in 4% paraformaldehyde, the duodenal, jejunal, and ileal segments were dehydrated through a graded ethanol series and subsequently embedded in paraffin. Serial sections (5 μm) were cut using a rotary microtome (RM2235, Leica Microsystems, Wetzlar, Germany) and stained with hematoxylin and eosin for histological evaluation. Morphological images were captured with a DMi8 inverted microscope (Leica Microsystems) equipped with a digital camera, and villus height (VH) and crypt depth (CD) were quantified using ImageJ software (version 2.16.0, National Institutes of Health, Bethesda, MD, USA).

### 2.10. Intestinal Microbiota Analysis

Total microbial DNA was extracted from cecal contents of Danzhou chickens using the E.Z.N.A.^®^ Stool DNA Kit (Omega Bio-tek, Norcross, GA, USA). DNA concentration was measured with the Quant-iT PicoGreen dsDNA assay (Invitrogen, Carlsbad, CA, USA) on a Quantus fluorometer (Promega, Madison, WI, USA), and extraction quality was verified by 1% agarose gel electrophoresis. The V3–V4 hypervariable region of the 16S rRNA gene was amplified with primers 515F (5′-barcode-GTGCCAGCMGCCGCGG-3′) and 907R (5′-CCGTCAATTCMTTTRAGTTT-3′). PCR amplicons were separated by 2% agarose gel electrophoresis, target bands were excised and purified using the AP-GX-250 AxyPrep DNA Gel Extraction Kit (Axygen, Corning, NY, USA), followed by Tris-HCl elution and magnetic bead purification. Libraries were constructed with the TruSeq DNA Library Prep Kit (Illumina, San Diego, CA, USA) and sequenced (2 × 250 bp paired-end) on an Illumina MiSeq PE250 platform using the MiSeq v2 reagent kit (Illumina). The obtained Illumina paired-end sequencing data were processed in the QIIME 2 environment (version 2019.4) [22]. Primers were first removed from the reads using the cutadapt plugin (version 2.3). After quality filtering, high-quality sequences were clustered into operational taxonomic units (OTUs) at a 97% similarity threshold. Taxonomic annotation of representative OTU sequences was performed using the UCLUST algorithm against the SILVA 138 database with an 80% confidence threshold. Features with classification confidence below 80% were assigned to the last confidently identified taxonomic level followed by the suffix “_unidentified”. Microbial community diversity analysis was conducted using the phyloseq package (version 1.34.0) in R software (version 4.0.3). Alpha diversity was assessed using indices including Chao1 and Shannon, among others. Beta diversity was calculated based on unweighted UniFrac distance and visualized through principal coordinate analysis (PCoA). Analysis of similarities (ANOSIM) with 999 permutations was employed to evaluate the statistical significance of differences in microbial community structure between groups. Differentially abundant taxa across groups were identified using linear discriminant analysis effect size (LEfSe), with an LDA score threshold set at >3.0 and a *p*-value < 0.05.

### 2.11. Statistical Analysis

Data were analyzed using SPSS 26.0 statistical software and are presented as mean and standard error of the mean (SEM). All data were first subjected to normality and homogeneity of variances tests, followed by two-way ANOVA to evaluate the main effects of BC and TB as well as their interaction. The statistical significance level was set at *p* < 0.05. The model was as follows:

*Y_ij_* = *μ* + *α_i_* + *β_j_* + *α_i_* × *β_j_* + *e_ij_*,

where *Y_ij_* is the observed value under BC *i* and TB *j*; *μ* is the overall mean; *α_i_* is the fixed effect of BC (*i* = 1, 2); *β_j_* is the fixed effect of TB (*j* = 1, 2); *α_i_* × *β_j_* is the interaction between BC and TB; and *e_ij_* is the random residual. When the interaction between BC and TB was significant, independent samples *t*-test was used to compare data across different supplementation levels of BC and TB, respectively. When the interaction was not significant, treatment groups were compared in the pooled data. A significant “BC effect” or “TB effect” was reported as a “main effect”.

## 3. Results

### 3.1. Growth Performance

The effects of different dietary treatments on growth performance of Danzhou chickens are summarized in Table 2. ADFI was not significantly influenced by any of the dietary treatments (*p* > 0.05). Supplementation with either 1.5 g/kg BC or 1 g/kg TB significantly increased the final body weight (FBW) and improved the FCR (*p* < 0.05). Furthermore, a significant interaction between BC and TB was observed for ADG (*p* < 0.05). Simple effect analysis revealed that the inclusion of 1.5 g/kg BC in the diet significantly enhanced ADG regardless of the TB supplementation level (0 or 1 g/kg) (*p* < 0.05). Similarly, the addition of 1 g/kg TB also significantly increased ADG, and this effect was consistent across both BC supplementation levels (*p* < 0.05).

### 3.2. Serum Biochemical Indicators

As shown in Table 3, dietary supplementation with 1.5 g/kg BC significantly increased serum ALB content and decreased AN content in Danzhou chickens (*p* < 0.05). A significant interaction was observed between BC and TB on TG and NEFA levels (*p* < 0.05). When the diet was supplemented with 1.5 g/kg BC, the addition of 1 g/kg TB significantly increased serum TG and NEFA concentrations (*p* < 0.05). Meanwhile, under the condition of 1 g/kg TB supplementation, the addition of 1.5 g/kg BC also significantly elevated serum TG levels (*p* < 0.05). No significant differences were observed in the remaining serum biochemical indicators among the different treatment groups (*p* > 0.05).

### 3.3. Immune Performance

As shown in Table 4, different dietary treatments significantly affected the immune organ indices of Danzhou chickens. Supplementation with BC and TB significantly increased the spleen index and bursa index (*p* < 0.05), while no significant change was observed in the bursa of bursa index (*p* > 0.05). Furthermore, dietary BC and TB significantly elevated serum levels of IgA, IgG, IL-4, and IL-10, and reduced the serum concentration of IL-6 (*p* < 0.05). A significant interaction was observed between BC and TB on serum levels of IgM, IL-1β, and TNF-α (*p* < 0.05). Specifically, when the supplementation level of TB (0 or 1 g/kg) was fixed, the addition of 1.5 g/kg BC significantly decreased serum IL-1β and TNF-α levels and increased IgM concentration (*p* < 0.05). Similarly, when the BC supplementation level (0 or 1.5 g/kg) was fixed, the addition of 1 g/kg TB also significantly reduced serum IL-1β and TNF-α levels (*p* < 0.05). However, the increase in IgM levels was observed only when 1.5 g/kg BC was supplemented, with a further addition of 1 g/kg TB producing a significant enhancement (*p* < 0.05).

### 3.4. Serum Antioxidant Indexes

As shown in Table 5, dietary supplementation with 1.5 g/kg BC and 1 g/kg TB significantly increased serum levels of TAOC and CAT in Danzhou chickens (*p* < 0.05). Furthermore, a significant interaction was observed between BC and TB on serum SOD activity (*p* < 0.05). Specifically, when the basal diet contained no BC, the addition of 1 g/kg TB significantly enhanced serum SOD levels (*p* < 0.05). In contrast, when 1.5 g/kg BC was supplemented, the inclusion of TB did not lead to any significant change in SOD activity (*p* > 0.05). Similarly, in the absence of TB, supplementation with 1.5 g/kg BC significantly increased serum SOD concentration (*p* < 0.05), whereas no significant effect was observed when the diet already contained 1 g/kg TB (*p* > 0.05).

### 3.5. Intestinal Digestive Enzyme Activities

As shown in Table 6, both BC and TB significantly increased amylase activity in the duodenum (*p* < 0.05), with no significant interaction observed between the two factors. In contrast, a significant interaction between BC and TB was detected for lipase activity in the ileum (*p* < 0.05). Specifically, the combined supplementation of 1.5 g/kg BC and 1 g/kg TB significantly enhanced ileal lipase activity compared to the individual supplementation of either 1.5 g/kg BC or 1 g/kg TB alone (*p* < 0.05). No significant differences were observed in the activities of other digestive enzymes across different intestinal segments among the treatment groups (*p* > 0.05).

### 3.6. Intestinal Morphology

As shown in Table 7, dietary supplementation with 1.5 g/kg BC significantly increased the villus height to crypt depth (VH/CD) ratio in the duodenum and jejunum, while reducing CD in the jejunum of Danzhou chickens (*p* < 0.05). In addition, supplementation with either BC or TB significantly increased VH in the jejunum and ileum (*p* < 0.05). Significant interactions between TB and BC were observed for duodenal VH and CD, as well as for ileal CD and the VH/CD (*p* < 0.05). Specifically, in the absence of BC, the addition of 1 g/kg TB significantly increased the ileal VH/CD and decreased ileal CD (*p* < 0.05). When the diet was supplemented with 1.5 g/kg BC, the addition of 1 g/kg TB significantly increased duodenal VH and the ileal VH/CD (*p* < 0.05). Under the condition of 1 g/kg TB supplementation, the addition of 1.5 g/kg BC significantly increased duodenal VH and ileal CD, but decreased the ileal VH/CD (*p* < 0.05). Similarly, in the absence of TB, supplementation with 1.5 g/kg BC also significantly increased duodenal VH (*p* < 0.05).

### 3.7. Diversity Changes in Gut Microbiota

**Figure 1:**
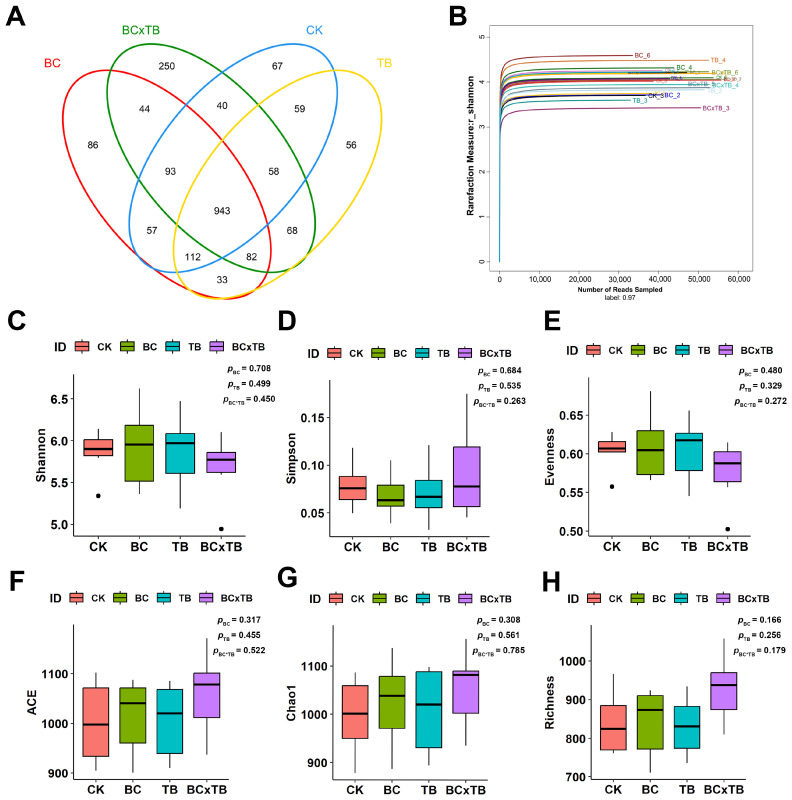
Effects of dietary *Bacillus coagulans* and tributyrin supplementation on the cecal microbiota composition of Danzhou chickens (*n* = 6). (**A**) Venn diagram of cecal microorganisms in each treatment group. (**B**) Rarefaction Curve based on shannon index. (**C**) Shannon index of cecal microbiota. (**D**) Simpson index of cecal microbiota. (**E**) Evenness index of cecal microbiota. (**F**) ACE index of cecal microbiota. (**G**) Chao1 index of cecal microbiota. (**H**) Richness index of cecal microbiota. CK = the basal diet; BC = the basal diet supplemented with 1.5 g/kg *Bacillus coagulans*; TB = the basal diet supplemented with 1.0 g/kg tributyrin; BC × TB = the basal diet supplemented with 1.5 g/kg *Bacillus coagulans* and 1.0 g/kg tributyrin.

High-throughput sequencing of the 16S rRNA gene was employed to investigate the effects of dietary supplementation with BC and TB on the cecal microbiota of Danzhou chickens. At a 97% sequence similarity threshold, a total of 2048 operational taxonomic units (OTUs) were identified across the four experimental groups: CK, BC, TB, and BC × TB (Figure 1A). Among these, the numbers of unique OTUs specific to each group were 67, 86, 56, and 250, respectively, while 943 core OTUs were shared by all four groups. Rarefaction curves based on the Shannon index gradually plateaued (Figure 1B), confirming that sufficient sequencing depth had been achieved to adequately characterize microbial diversity and community composition. Compared to the CK group, the BC × TB group exhibited increased α-diversity indices (including Chao1, ACE, and Richness); however, these differences did not reach statistical significance (Figure 1C–H, *p* > 0.05).

### 3.8. Compositional Changes in Gut Microbiota

**Figure 2:**
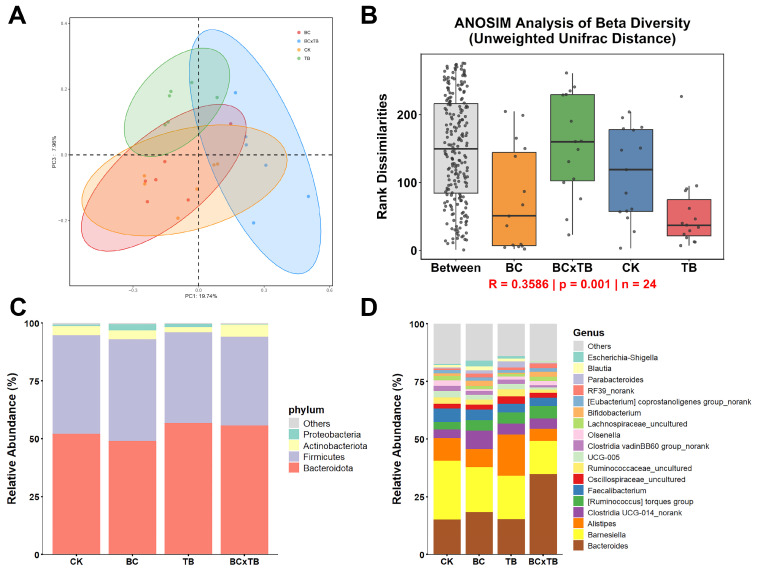
Effects of dietary *Bacillus coagulans* and tributyrin supplementation on the cecal microbiota composition of Danzhou chickens (*n* = 6). (**A**) Cecal microbial beta diversity. (**B**) ANOSIM analysis of beta diversity. Major cecal microbiota at the phylum (**C**) and genus (**D**) levels. Bacterial taxa with an average relative abundance of less than 1% were consolidated into the ‘Others’ category. CK = the basal diet; BC = the basal diet supplemented with 1.5 g/kg *Bacillus coagulans*; TB = the basal diet supplemented with 1.0 g/kg tributyrin; BC × TB = the basal diet supplemented with 1.5 g/kg *Bacillus coagulans* and 1.0 g/kg tributyrin.

Based on unweighted UniFrac distance, PCoA revealed that the first principal component explained 19.74% of the variation, while the third principal component explained 7.96%. A clear separation was observed between the CK group and both the BC and BC × TB groups, indicating that dietary supplementation with TB or a combination of TB and BC led to significant differences in microbial composition (Figure 2A). This observation was reinforced by ANOSIM analysis (Figure 2B), which confirmed that differences between groups were greater than those within groups, demonstrating significant distinctions in microbial communities among the different treatment groups (R = 0.359, *p* = 0.001). Evaluation of the effects of dietary TB and BC on the representation of different bacterial taxa showed that the dominant phyla in each treatment group were *Bacteroidota*, *Firmicutes*, *Actinobacteriota*, and *Proteobacteria*. *Bacteroidota* was the most abundant phylum, with its relative abundance lower in the BC group compared to the control group (CK), while it increased in the TB and TB × BC groups (Figure 2C). Furthermore, the relative abundance of *Firmicutes* was higher in the BC group compared to the CK group, whereas that of *Actinobacteriota* decreased in the TB group. At the genus level, *Barnesiella* was dominant in the CK group but its relative abundance decreased in the treatment groups. Additionally, compared to the CK group, the relative abundances of *Bacteroides*, *Alistipes*, and *Clostridia UCG-014_norank* increased in the BC × TB, TB, and BC groups, respectively. The relative abundance of *Oscillospiraceae_uncultured* increased in the TB group, while that of *Ruminococcaceae_uncultured* decreased in the BC × TB group. Meanwhile, the relative abundances of *UCG-005* and *Clostridia_vadinBB60_group_norank* decreased across all treatment groups (Figure 2D).

### 3.9. Impact of Dietary Bacillus coagulans and Tributyrin Supplementation on the Gut Microbiota via Lefse Analysis

**Figure 3:**
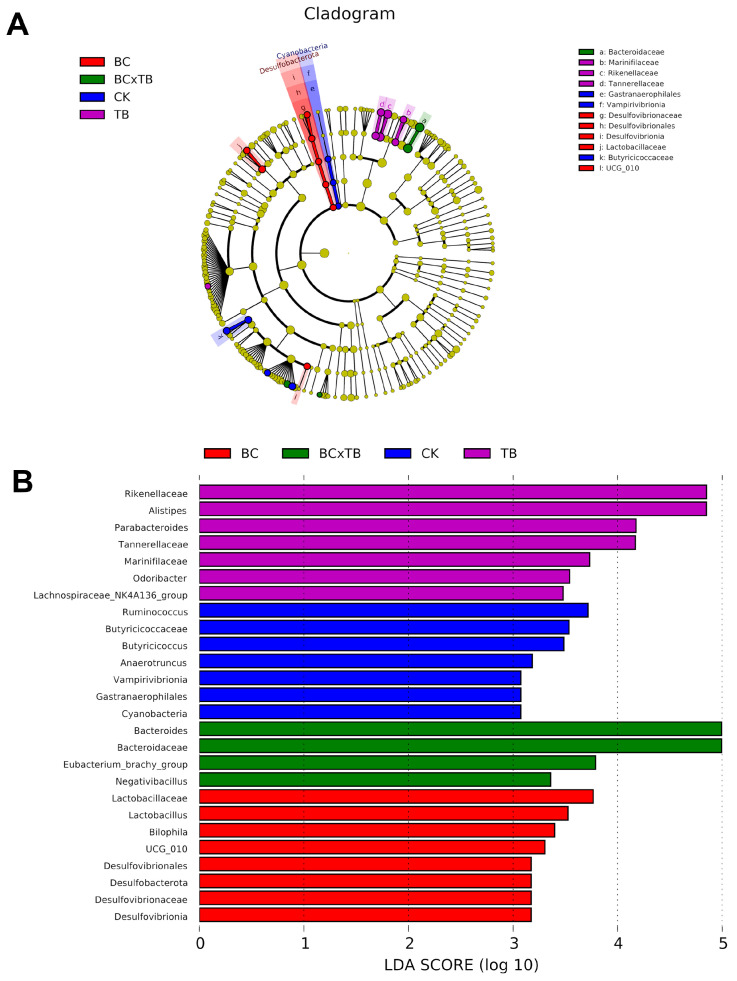
Effects of dietary *Bacillus coagulans* and tributyrin supplementation on the cecal microbiota composition of Danzhou chickens (*n* = 6). (**A**) Phylogenetic tree derived from LEfSe analysis. (**B**) LDA histograms. CK = the basal diet; BC = the basal diet supplemented with 1.5 g/kg *Bacillus coagulans*; TB = the basal diet supplemented with 1.0 g/kg tributyrin; BC × TB = the basal diet supplemented with 1.5 g/kg *Bacillus coagulans* and 1.0 g/kg tributyrin.

LEfSe analysis incorporating Linear Discriminant Analysis (LDA) identified differentially abundant bacterial taxa and classified them according to effect size (Figure 3A,B). Using a threshold of *p* < 0.05 and LDA > 3, 26 significantly enriched taxonomic features were detected from phylum to species level. At the phylum level, *Cyanobacteria* and *Desulfobacterota* were significantly enriched in the CK and BC groups, respectively. Analysis at the genus level revealed significant enrichment of *Ruminococcus*, *Butyricicoccus*, and *Anaerotruncus* in the CK group (*p* < 0.05), while *Lactobacillus*, *Bilophila*, and *UCG_010* were enriched in the BC group (*p* < 0.05). The TB group showed enrichment of *Alistipes*, *Parabacteroides*, *Odoribacter*, and *Lachnospiraceae_NK4A136_group* (*p* < 0.05). Notably, *Bacteroides*, *Eubacterium_brachy_group*, and *Negativibacillus* were significantly enriched in the BC × TB group (*p* < 0.05).

### 3.10. Correlation Analysis Between Gut Microbiota and Antioxidant and Immune Performance

**Figure 4:**
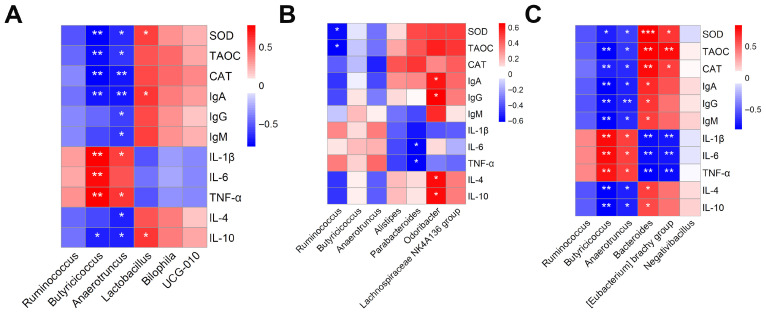
Correlation analysis between gut microbiota and antioxidant and immune performance (*n* = 6). (**A**) Correlation analysis of gut microbiota with antioxidant and immune indices in the CK versus BC groups. (**B**) Correlation analysis of gut microbiota with antioxidant and immune indices in the CK versus TB groups. (**C**) Correlation analysis of gut microbiota with antioxidant and immune indices in the CK versus BC × TB groups. CK = the basal diet; BC = the basal diet supplemented with 1.5 g/kg *Bacillus coagulans*; TB = the basal diet supplemented with 1.0 g/kg tributyrin; BC × TB = the basal diet supplemented with 1.5 g/kg *Bacillus coagulans* and 1.0 g/kg tributyrin. * indicates *p* < 0.05; ** indicates *p* < 0.01; *** indicates *p* < 0.001.

To investigate the relationship between gut microbiota and immune and antioxidant performance in Danzhou chickens, we conducted Pearson correlation analyses for each group (Figure 4A–C). The results revealed that in comparisons of the BC and BC × TB groups with the CK group, *Butyricicoccus* and *Anaerotruncus* showed significant negative correlations with serum antioxidant indices (SOD, TAOC, CAT), immune indices (IgA), and the anti-inflammatory cytokine IL-10, while exhibiting significant positive correlations with pro-inflammatory cytokines (IL-1β, TNF-α) (*p* < 0.05). Additionally, in the analysis of the BC group versus the CK group, *Lactobacillus* was positively correlated with SOD, IL-10, and IgA (*p* < 0.05). In the correlation analysis of the BC × TB group with the CK group, *Bacteroides* was positively correlated with antioxidant indices, immune indices, and anti-inflammatory cytokines, and negatively correlated with pro-inflammatory cytokines (*p* < 0.05), while *Eubacterium_brachy_group* was positively correlated only with antioxidant indices and negatively correlated with pro-inflammatory cytokines (*p* < 0.05). Further analysis of the TB group versus the CK group indicated that *Parabacteroides* was negatively correlated with pro-inflammatory cytokines (IL-6, TNF-α) (*p* < 0.05), while *Odoribacter* was positively correlated with IL-4, IL-10, IgA, and IgG (*p* < 0.05).

## 4. Discussion

Growth performance serves as a critical indicator of poultry production efficiency. BC, a clean and safe probiotic additive, has been demonstrated to enhance growth performance in livestock and poultry [23]. Previous studies in broilers have demonstrated that dietary supplementation with BC enhances body weight gain, average daily gain, and feed conversion efficiency [8,24]. Likewise, TB, a derivative of butyrate, also exerts beneficial effects on broiler growth and health. Research by Ismael et al. [12] confirmed that adding 300 g/ton of a TB-based mixture to the diet significantly increased FBW and the European production efficiency factor. Consistent with these earlier findings, the present experiment revealed that individual supplementation with either TB or BC significantly increased the FBW and ADG, while reducing the FCR in Danzhou chickens. Moreover, this study demonstrated that the combined supplementation of TB and BC resulted in a more pronounced improvement in ADG than the use of either additive alone, which aligns with the conclusions reported by Hou et al. [15] in yellow-feathered broilers. This synergistic effect is likely attributable to their complementary modes of action: BC primarily maintains the balance of beneficial intestinal microbiota and promotes feed digestion and nutrient absorption [25], whereas TB increases the abundance of short-chain fatty acid–producing bacteria and directly stimulates butyrate production in the gut, thereby jointly contributing to more efficient growth promotion [26].

Blood biochemical parameters are essential indicators of nutrient digestion and absorption, as well as overall health status in poultry [27]. The results of this study demonstrated that dietary supplementation with BC improved the blood biochemical profile of Danzhou chickens. As a probiotic, BC colonizes the intestine and secretes various active enzymes that promote the digestion, absorption, and synthesis of nutrients such as proteins and short-chain fatty acids [28,29]. Consistent with previous studies [30], supplementation with BC significantly increased serum TP and ALB levels, indicating enhanced protein metabolism. Meanwhile, AN levels serve as a common indicator of dietary protein utilization efficiency. This study found that BC supplementation significantly reduced serum AN concentrations, suggesting improved dietary protein utilization. Furthermore, the combined supplementation of BC and TB significantly increased serum TG and NEFA levels, which may be associated with their synergistic effect on promoting intestinal digestive enzyme activity. Specifically, the combined treatment significantly enhanced amylase activity in the duodenum and lipase activity in the ileum. Previous studies have shown that BC can produce digestive enzymes such as amylase, lipase, and protease [31], while TB also enhances digestive enzyme activity [32]. Therefore, the combination of these two additives appears to enhance intestinal digestive enzyme activity, thereby improving nutrient digestion and absorption, which consequently elevates serum TG and NEFA levels.

Dynamic oxidative balance serves as a critical indicator of poultry health. An imbalance between the oxidative and antioxidant systems within the body can induce oxidative stress, resulting in the excessive generation of reactive oxygen species (ROS) such as hydroxyl radicals and superoxide anions [33]. These ROS molecules damage biological macromolecules including proteins and nucleic acids, thereby causing tissue and mitochondrial injury [34]. Under modern intensive farming conditions, heat stress further aggravates ROS production, leading to oxidative stress–related damage in broilers [35]. Antioxidant enzymes such as SOD and CAT play a crucial role in mitigating oxidative stress and neutralizing ROS. Previous research has found that BC can enhance the antioxidant capacity of laying hens by increasing T-SOD activity and reducing serum MDA concentration [36], while TB can elevate the T-AOC in broilers [37]. Consistent with these findings, the present study demonstrated that both BC and TB supplementation improved the antioxidant status of Danzhou chickens by elevating serum levels of SOD, T-AOC, and CAT. In conclusion, dietary supplementation with either BC or TB can effectively mitigate oxidative stress–induced damage, which likely contributes to the enhanced growth performance observed in Danzhou chickens.

Modern broiler production is highly susceptible to environmental and external stressors, with immune development closely linked to growth performance, morbidity, and mortality. Immune organ indices serve as important indicators for assessing the immune status of poultry. The bursa of Fabricius, thymus, and spleen are key immune organs in birds [38]. As an avian-specific organ, the bursa of Fabricius is highly sensitive to dietary compositional changes and serves as the primary site for B lymphocyte development and differentiation, directly regulating humoral immunity [39]. Previous studies have shown that dietary supplementation with BC increased the relative weight of the bursa in broilers [40], while TB supplementation improved the spleen index in weaned piglets [41]. Similarly, in this study, dietary supplementation with both BC and TB significantly increased the spleen and bursa indices in Danzhou chickens. Since immunoglobulins such as IgA, IgG, and IgM are produced in lymphoid tissues after B cells are stimulated by antigens or allergens and are key components of humoral immunity [42], well-developed bursa facilitates immunoglobulin synthesis. Consistent with this, the present study found that supplementing the diet with BC and TB significantly elevated serum levels of IgA, IgG, and IgM in Danzhou chickens, with the combined supplementation showing the most pronounced effect on IgM levels. This aligns with previous reports that both BC and TB can increase serum immunoglobulin levels in livestock and poultry [43,44]. Furthermore, cytokines, as essential components of innate immunity, also play a critical role in immune regulation. This study further demonstrated that supplementation with BC and TB significantly elevated serum levels of anti-inflammatory cytokines (IL-10, IL-4) and reduced pro-inflammatory cytokines (IL-1β, IL-6, TNF-α), with the combined supplementation most effectively reducing IL-1β and TNF-α. These findings are consistent with Zhang et al.’s [44] report in broilers, where oral administration of BC at 10^8 CFU/mL enhanced anti-inflammatory effects and reduced mRNA expression of the pro-inflammatory cytokines IL-1β and TNF-α, and TB showed similar anti-inflammatory effects in fish studies [45]. In conclusion, dietary supplementation with BC and TB significantly enhanced the immune performance of Danzhou chickens, and their combined use was more effective than individual supplementation.

The small intestine serves as the primary site for nutrient digestion and absorption, with intestinal villi being the key structures responsible for performing absorptive functions. VH, CD, and their ratio (VH/CD) are closely associated with intestinal digestion, absorption, and cell maturation and differentiation [6]. Generally, greater VH, shallower CD, and a higher VH/CD ratio indicate enhanced digestive and absorptive capacity, as well as a more mature mucosal structure [46]. BC, as a probiotic, has been demonstrated to improve the intestinal morphology of livestock and poultry. Previous studies have shown that supplementing broiler diets with 1 × 10^9^ CFU/kg of BC significantly increased VH and the VH/CD ratio in the duodenum and ileum [47]; similarly, supplementation with 6.5 × 10^5^ CFU/g also significantly improved the VH/CD ratio in the ileum [33]. In addition, butyrate plays an important role in promoting intestinal development and enhancing mucosal architecture. TB, as a source of butyric acid, not only directly supplies butyrate but also promotes villus growth and development by enhancing cellular anabolic activity and upregulating the expression of the butyrate transporter MCT-1 [11]. Research by Hu et al. [48] further confirmed this, demonstrating that dietary supplementation with TB significantly increased VH and the VH/CD ratio in the intestines of broilers. Consistent with these findings, the present study found that dietary supplementation with BC increased VH and the VH/CD ratio in the duodenum and jejunum, as well as VH in the ileum of Danzhou chickens, while TB treatment enhanced VH throughout the small intestine and the VH/CD ratio in the ileum. Moreover, the combined supplementation of BC and TB further enhanced duodenal villus height, indicating a potential synergistic effect on intestinal villus development.

The gut microbiota plays a fundamental role in maintaining broiler health and promoting production efficiency through its effects on nutrient absorption, growth performance, and immune system modulation [49,50]. Previous research in broilers has shown that dietary supplementation with TB alone increases the abundance of beneficial bacteria such as *Lactobacillus* and *Bacillus* while inhibiting *Salmonella* colonization [48,51]. Similarly, supplementation with BC alone has been found to elevate beneficial bacteria like *Alistipes* and *Odoribacter* and reduce harmful bacteria such as *Desulfovibrio* and *Parasutterella* [44]. These findings are consistent with the present study, in which individual or combined supplementation with BC or TB generally shifted the microbial community toward a more beneficial composition. Specifically, all treatment groups reduced the abundance of *Anaerotruncus*—which is enriched in the control group and positively correlated with inflammatory responses [52]. Supplementation with BC alone increased the abundance of *Lactobacillus* and *UCG_010*, while TB alone elevated the abundance of *Parabacteroides*, *Odoribacter*, and *Lachnospiraceae_NK4A136_group*. *Lactobacillus* enhances antioxidant capacity, reduces inflammation, and improves the microvillus structure of intestinal epithelial cells in broilers [53], which aligns with the positive correlations observed in this study between Lactobacillus and the serum antioxidant indicator SOD as well as the anti-inflammatory cytokine IL-10. Butyrate-producing bacteria such as *UCG_010*, *Lachnospiraceae_NK4A136_group*, and *Odoribacter* play important roles in improving intestinal morphology, maintaining barrier integrity, and promoting growth performance [54,55,56,57]. *Parabacteroides* has also been demonstrated to exert beneficial effects on intestinal inflammation and enhance gut barrier function [58], further supported by the negative correlation between Parabacteroides and pro-inflammatory cytokines found in this study. These microbial shifts explain the previously observed improvements in antioxidant status and growth performance, along with reduced inflammation, in broilers supplemented individually with either TB or BC. However, individual supplementation with BC or TB also resulted in the enrichment of *Bilophila* and *Alistipes*, respectively, both of which possess pro-inflammatory potential [56,59]. This issue was resolved by their combined use, which exclusively promoted the abundance of beneficial genera such as *Bacteroides*, *Eubacterium_brachy_group*, and *Negativibacillus*. Previous studies indicate that *Bacteroides* helps reduce oxidative stress and inflammation while enhancing immune function [60], which is consistent with the correlation analysis results of this study, whereas *Eubacterium_brachy_group* and *Negativibacillus* contribute primarily to improved feed efficiency and growth performance [61,62]. These findings further support a synergistic interaction between TB and BC and may explain why the combined treatment group (BC × TB) exhibited the best overall growth performance, antioxidant capacity, and immune response. In conclusion, the combined supplementation more effectively modulated the gut microbiota toward a healthier metabolic profile.

## 5. Conclusions

This study demonstrates that dietary supplementation with 1.5 g/kg BC or 1 g/kg TB alone significantly improved growth performance, antioxidant capacity, and immune function in Danzhou chickens, while also enhancing intestinal morphology and increasing the richness and diversity of the cecal microbiota. Furthermore, supplementation with BC or TB promoted increased protein and lipid levels in serum by enhancing digestive enzyme activity, thereby supporting improved nutrient absorption and growth. More importantly, the combined use of BC and TB exhibited significant synergistic effects across multiple physiological metrics, resulting in optimal outcomes in terms of growth performance, antioxidant capacity, immune response, intestinal morphology, and gut microbiota. Therefore, the combined supplementation of 1.5 g/kg BC and 1 g/kg TB could serve as an effective antibiotic-alternative strategy for green feeding in poultry production.

## Figures and Tables

**Table 1:** Composition and nutrient levels of the basal diet (as dry basis, %).

Ingredients, %	Content	Nutrient Levels ^2^	Content
Corn	21.30	ME, MJ/kg	12.10
Wheat	43.64	Crude protein, %	21.00
Soybean meal	19.26	Crude fiber, %	2.96
Lard	1.67	Ca, %	0.88
CaHPO_4_	1.15	Available phosphorus, %	0.35
Limestone	1.34	Lysine, %	1.15
Puffing of soybean	3.00	Methionine, %	0.52
Peanut meal	3.00		
Sunflower meal	2.00		
Corn glutenmeal	2.56		
NaHCO_3_	0.20		
NaCl	0.25		
Methionine	0.23		
Threonine	0.15		
Lysine sulfate	0.69		
Choline	0.08		
Premix ^1^	0.80		
Total	100.00		

^1^ Provided per kilogram of premix: VA 40,000 IU, VD3 12,000 IU, VE 145 mg, VK 1.0 mg, VB_1_ 2.4 mg, VB_2_ 5 mg, VB_3_ 40 mg, VB_5_ 15 mg, VB_6_ 5 mg, VB_7_ 0.25 mg, VB_11_ 1.4 mg, VB_12_ 0.02 mg, Mn 70 mg, I 0.7 mg, Fe 100 mg, Cu 10 mg, Zn 80 mg, Se 0.34 mg. ^2^ Metabolizable energy, lysine, and methionine levels are calculated values, and other nutrient levels are measured values. ME = metabolizable energy.

**Table 2:** Effects of dietary supplementation with BC and TB on the growth performance of Danzhou chickens (*n* = 6).

Items ^1^	Treatment ^2^				SEM	*p*-Value		
	BC, 0 g/kg		BC, 1.5 g/kg					
	TB, 0 g/kg	TB, 1 g/kg	TB, 0 g/kg	TB, 1 g/kg		BC	TB	BC × TB
IBW, g	24.75	24.58	24.55	24.62	0.60	0.896	0.935	0.845
FBW, g	360.05	383.33	381.20	431.32	9.23	0.001	0.001	0.370
ADFI, g/d	24.12	23.51	23.75	23.56	0.24	0.540	0.134	0.419
ADG, g/d	9.58 ^Bb^	10.25 ^Ab^	10.19 ^Ba^	11.62 ^Aa^	0.17	<0.001	<0.001	0.041
FCR	2.52	2.29	2.33	2.02	0.09	0.027	0.011	0.669

^1^ ADFI, average daily feed intake; ADG, average daily gain; FCR, feed conversion ratio. ^2^ BC, *Bacillus coagulans*; TB, tributyrin. A significant difference in values within the same row is indicated by different superscript letters. Uppercase letters (A, B) denote significant differences between TB treatments at a fixed level of BC (*p* < 0.05). Lowercase letters (a, b) denote significant differences between BC treatments at a fixed level of TB (*p* < 0.05).

**Table 3:** Effects of dietary supplementation with BC and TB on serum biochemical parameters of Danzhou chickens (*n* = 6).

Items ^1^	Treatment ^2^				SEM	*p*-Value		
	BC, 0 g/kg		BC, 1.5 g/kg					
	TB, 0 g/kg	TB, 1 g/kg	TB, 0 g/kg	TB, 1 g/kg		BC	TB	BC × TB
TP, g/L	19.51	23.65	24.75	23.97	1.48	0.083	0.284	0.123
ALB, g/L	8.82	10.2	11.3	10.65	0.62	0.034	0.581	0.133
GLB, g/L	10.68	13.46	13.45	13.32	0.95	0.206	0.205	0.165
TC, mmol/L	2.34	2.38	2.39	2.42	0.16	0.789	0.835	0.976
TG, mmol/L	0.48	0.50 ^b^	0.51 ^B^	0.72 ^Aa^	0.04	0.01	0.016	0.043
HDL, mmol/L	1.30	1.32	1.34	1.33	0.06	0.678	0.934	0.803
LDL, mmol/L	0.74	0.79	0.67	0.77	0.08	0.567	0.338	0.725
UREA, mmol/L	0.96	0.91	0.63	0.76	0.06	<0.001	0.594	0.143
UA, umol/L	237.77	234.61	246.75	258.43	31.13	0.627	0.899	0.826
AN, umol/L	21.48	20.74	20.21	20.49	0.4	0.077	0.579	0.225
NEFA, mmol/L	0.46	0.48	0.35 ^B^	0.65 ^A^	0.06	0.584	0.011	0.031
ALP, U/L	4128.83	3740.15	2505.05	2993.42	715.47	0.116	0.946	0.55

^1^ TC, total cholesterol; TG, triglycerides; HDL, high-density lipoprotein cholesterol; LDL, low-density lipoprotein cholesterol; UA, uric acid; AN, ammonia nitrogen; NEFA, non-esterified fatty acids; ALP, alkaline phosphatase. ^2^ BC, *Bacillus coagulans*; TB, tributyrin. A significant difference in values within the same row is indicated by different superscript letters. Uppercase letters (A, B) denote significant differences between TB treatments at a fixed level of BC (*p* < 0.05). Lowercase letters (a, b) denote significant differences between BC treatments at a fixed level of TB (*p* < 0.05).

**Table 4:** Effects of dietary supplementation with BC and TB on the immune performance of Danzhou chickens (*n* = 6).

Items ^1^	Treatment ^2^				SEM	*p*-Value		
	BC, 0 g/kg		BC, 1.5 g/kg					
	TB, 0 g/kg	TB, 1 g/kg	TB, 0 g/kg	TB, 1 g/kg		BC	TB	BC × TB
Spleen index, g/kg	1.17	1.53	1.49	1.68	0.09	0.025	0.010	0.390
Bursa index, g/kg	3.23	4.27	4.31	4.96	0.38	0.032	0.04	0.618
Thymus index, g/kg	3.07	3.34	3.27	3.38	0.3	0.696	0.533	0.795
IgA, g/L	1.81	2.47	2.35	2.96	0.05	<0.001	<0.001	0.645
IgG, g/L	4.41	5.42	5.32	5.82	0.20	0.005	0.002	0.235
IgM, g/L	1.54 ^b^	1.56 ^b^	1.71 ^Ba^	1.92 ^Aa^	0.04	<0.001	0.013	0.037
IL-1β, pg/mL	36.58 ^Aa^	29.84 ^Ba^	26.56 ^Ab^	23.42 ^Bb^	0.58	<0.001	<0.001	0.006
IL-6, pg/mL	234.50	219.12	185.13	159.61	5.57	<0.001	0.002	0.403
TNF-α, pg/mL	86.86 ^Aa^	74.86 ^Ba^	68.26 ^Ab^	62.95 ^Bb^	1.43	<0.001	<0.001	0.035
IL-4, pg/mL	5.19	7.38	6.75	8.95	0.32	<0.001	<0.001	0.992
IL-10, pg/mL	11.56	14.07	16.24	17.28	0.51	<0.001	0.003	0.174

^1^ IgA, immunoglobulin A; IgG, immunoglobulin G; IgM, immunoglobulin M; IL-1β, interleukin-1 beta; IL-6, interleukin-6; TNF-a, tumor necrosis factor-alpha; IL-4, interleukin-4; IL-10, interleukin-10. ^2^ BC, *Bacillus coagulans*; TB, tributyrin. A significant difference in values within the same row is indicated by different superscript letters. Uppercase letters (A, B) denote significant differences between TB treatments at a fixed level of BC (*p* < 0.05). Lowercase letters (a, b) denote significant differences between BC treatments at a fixed level of TB (*p* < 0.05).

**Table 5:** Effects of dietary supplementation with BC and TB on antioxidant capacity of Danzhou chickens (*n* = 6).

Items ^1^	Treatment ^2^				SEM	*p*-Value		
	BC, 0 g/kg		BC, 1.5 g/kg					
	TB, 0 g/kg	TB, 1 g/kg	TB, 0 g/kg	TB, 1 g/kg		BC	TB	BC × TB
SOD, U/ml	52.8 ^Bb^	75.02 ^A^	79.88 ^a^	82.84	2.23	<0.001	<0.001	0.001
TAOC, U/ml	7.96	9.57	10.74	12.46	0.19	<0.001	<0.001	0.786
CAT, U/ml	43.84	49.57	53.97	60.24	0.80	<0.001	<0.001	0.769

^1^ SOD, superoxide dismutase; TAOC, total antioxidant capacity; CAT, catalase. ^2^ BC, *Bacillus coagulans*; TB, tributyrin. A significant difference in values within the same row is indicated by different superscript letters. Uppercase letters (A, B) denote significant differences between TB treatments at a fixed level of BC (*p* < 0.05). Lowercase letters (a, b) denote significant differences between BC treatments at a fixed level of TB (*p* < 0.05).

**Table 6:** Effects of dietary supplementation with BC and TB on intestinal digestive enzyme activities of Danzhou chickens (*n* = 6).

Items	Treatment ^1^				SEM	*p*-Value		
	BC, 0 g/kg		BC, 1.5 g/kg					
	TB, 0 g/kg	TB, 1 g/kg	TB, 0 g/kg	TB, 1 g/kg		BC	TB	BC × TB
Duodenum								
Amylase, U/mg prot	3.48	4.48	3.9	6.49	0.56	0.046	0.005	0.179
Trypsin, U/mg prot	2538.31	2004.88	2680.48	2966.62	307.23	0.093	0.697	0.205
Lipase, U/g prot	12.94	8.42	20.03	17.12	4.4	0.123	0.458	0.871
Jejunum								
Amylase, U/mg prot	3.09	4.25	3.51	5.76	1.04	0.371	0.12	0.612
Trypsin, U/mg prot	1857.38	2100.63	2168.95	2212.97	378.51	0.585	0.711	0.797
Lipase, U/g prot	9.99	12.02	20.94	19.96	6.85	0.215	0.944	0.84
Ileum								
Amylase, U/mg prot	4.05	4.00	2.48	4.01	0.94	0.457	0.48	0.449
Trypsin, U/mg prot	1932.97	1749.03	2378.45	2585.6	348.04	0.083	0.974	0.584
Lipase, U/g prot	16.44	16.48 ^b^	22.64 ^B^	42.99 ^Aa^	4.45	0.002	0.042	0.042

^1^ BC, *Bacillus coagulans*; TB, tributyrin. A significant difference in values within the same row is indicated by different superscript letters. Uppercase letters (A, B) denote significant differences between TB treatments at a fixed level of BC (*p* < 0.05). Lowercase letters (a, b) denote significant differences between BC treatments at a fixed level of TB (*p* < 0.05).

**Table 7:** Effects of dietary supplementation with BC and TB on intestinal morphology of Danzhou chickens (*n* = 6).

Items ^1^	Treatment ^2^				SEM	*p*-Value		
	BC, 0 g/kg		BC, 1.5 g/kg					
	TB, 0 g/kg	TB, 1 g/kg	TB, 0 g/kg	TB, 1 g/kg		BC	TB	BC × TB
Duodenum								
Villus height, μm	893 ^b^	911 ^b^	1019 ^Ba^	1220 ^Aa^	34.47	<0.001	0.018	0.044
Crypt depth, μm	195 ^a^	148	130 ^b^	157	13.2	0.05	0.466	0.012
VH/CD	4.58	6.14	7.81	7.73	0.67	0.002	0.295	0.247
Jejunum								
Villus height, μm	723	779	811	931	30.41	<0.001	0.010	0.311
Crypt depth, μm	180	208	120	131	11.2	<0.001	0.133	0.502
VH/CD	4.03	4.36	6.92	6.62	0.48	<0.001	0.976	0.538
Ileum								
Villus height, μm	489	696	554	768	16.36	<0.001	<0.001	0.836
Crypt depth, μm	162 ^A^	105 ^Bb^	161	168 ^a^	11.38	0.015	0.044	0.012
VH/CD	3.02 ^B^	6.61 ^Aa^	3.43 ^B^	4.57 ^Ab^	0.41	0.077	<0.001	0.011

^1^ VH/CD, villus height to crypt depth ratio. ^2^ BC, *Bacillus coagulans*; TB, tributyrin. A significant difference in values within the same row is indicated by different superscript letters. Uppercase letters (A, B) denote significant differences between TB treatments at a fixed level of BC (*p* < 0.05). Lowercase letters (a, b) denote significant differences between BC treatments at a fixed level of TB (*p* < 0.05).

## Data Availability

Data presented are original and not inappropriately selected, manipulated, enhanced, or fabricated. Raw and processed reads about 16S rDNA have been deposited in the NCBI Sequence Read Archive under BioProject PRJNA1356494.

## Supplementary Materials

The following supporting information can be downloaded at https://www.mdpi.com/article/10.3390/ani15233428/s1, Table S1: Detailed information of immunological performance kits; Table S2: Detailed information of antioxidant reagent kits.
