# Supplementary material for: Supplementation of Bacillus coagulans and Tributyrin to Danzhou Chickens: Effects on Growth Performance, Antioxidant Status, Immune Response, Intestinal Health, and Cecal Microbiome
Source: Animals (Basel). 2025 Nov 27;15(23):3428. doi: 10.3390/ani15233428 (PMC12691401; doi:10.3390/ani15233428)
Supplement: Supplementary file 1 [file animals-15-03428-s001.zip › animals-3994695-supplementary.pdf]

Table S1 Detailed information of immunological performance kits.

Item	Detection Range	Within-Batch Coefficient of Variation, %	Between-Batch Coefficient of Variation, %	Sensitivity
IgA, g/L	0-10	5.00	10.00	0.12
IgG, g/L	0-10	10.00	10.00	0.18
IgM, g/L	0-10	10.00	10.00	0.06
IL-1β, pg/ml	10-160	12.00	13.00	0.30
IL-6, pg/ml	50-800	4.50	8.00	3.00
TNF-α, pg/ml	12.5-200	5.00	8.00	0.30
IL-4, pg/ml	2-32	8.00	15.00	0.03
IL-10, pg/ml	5-80	8.00	15.00	0.50

Table S2 Detailed information of antioxidant reagent kits.

Items	Detection Range	Within-Batch Coefficient of Variation, %	Between-Batch Coefficient of Variation, %	Sensitivity
T-AOC, U/ml	0-15	7.30	10	0.20
SOD, U/ml	0-100	8.30	10	0.20
CAT, U/L	0-80	7.30	10	1.00
